# Editorial: Antifungal Resistance: From Molecular to Global Issues

**DOI:** 10.3389/fcimb.2022.867398

**Published:** 2022-03-02

**Authors:** Daisuke Hagiwara, Sabine Fillinger, Dominique Sanglard

**Affiliations:** ^1^ Faculty of Life and Environmental Sciences, University of Tsukuba, Tsukuba, Japan; ^2^ Université Paris-Saclay, INRAE, UR BIOGER, Saint Aubin, France; ^3^ Institute of Microbiology, University Hospital, University of Lausanne, Lausanne, Switzerland

**Keywords:** antifungal use, resistance mechanisms, animal and plant pathogenic fungi, drug discovery, drug efflux pump

Efficient antifungal compounds to be applied in the clinics or in agriculture are becoming rare, as resistance is increasing in the targeted populations. The case of the sterol demethylation inhibitors is particular, as this type of antifungal compounds corresponds to a common mode of action in the medical and the agricultural sector. All antifungal compounds are facing resistance issues leading to treatment failures and potentially death in the clinics, to treatment failure, yield loss and potential ecotoxicological problems due to additional treatments in agriculture. This Research Topic covered several aspects at the frontier between agriculture and the clinics.

Jørgensen and Heick comprehensively reviewed the use of azoles in agriculture, including protection of major crops and turfgrass as well as wood preservation. Azole fungicides undoubtedly play a significant role in the control of major diseases in a vast range of crops for more than 40 years. This has contributed to increasing yield in production and wider food supplies. Despite their significant importance, azole fungicides have been challenged by resistance issues at the same period. The authors highlighted the fact that azole resistance has been reported in 30 plant pathogens in over 60 countries. They also mentioned potential risk impacting human health through the emergence and spread of azole-resistant isolates of *A. fumigatus*.

Rocchi et al. addressed the question of links between patient infections, their potential acquisition from local environmental sources due to the shared mode of action between medical and agricultural antifungal compounds. They performed a large-scale genotyping analysis using microsatellites in *Aspergillus fumigatus* isolated in France, including 34 clinical and 191 environmental isolates. Among them, 29 and 84 strains were resistant to azoles, respectively. They were not able to rule out the possibility of some nosocomial transmission as genotypes were diverse even in a set of isolates from the same environment, suggesting a highly mixed azole-resistant *A. fumigatus* population. These data emphasized the importance of incorporating spatial sampling to understand the genetic structure of the local population of *A. fumigatus*.

Nagy et al. focused on pleiotropic drug resistance (PDR) as resistance mechanism against azoles in the human pathogenic fungus *Mucor circinelloides*. They performed functional analysis of the PDR transporter subfamily of ABC transporters and demonstrated that the regulation of the eight *pdr* genes is interconnected. The transporter genes *pdr1* and *pdr2* participate to the resistance of the fungus to azoles. Based on this finding, PDR-type transporters influence drug resistance in *Mucor* fungi as was reported in other pathogenic fungi such as *Candida* and *Aspergillus* species. This is the first report on molecular characterization of PDR-type transporter in Mucormycosis-related fungus.

Gaikani et al. undertook a study to identify new drug synergies based on a chemical-genetic analysis in *Saccharomyces cerevisiae*. They first performed the assessment of combinatorial effects of two compounds on the proliferation kinetics among a panel of 10 growth inhibitory compounds. Then, the eleven heterozygous deletion mutants, in which each known drug-target was deleted, were used to profile 121 drug-gene interaction tests. Eventually drug combinations were tested in an HIP HOP assay and probed for hypersensitive strains. In conclusion, 78% of the synergistic combinations tested were the result of combining an ergosterol inhibitor with a second agent. This report demonstrated a new strategy to predict drug synergy by using comprehensive genome-wide screens.

## Author Contributions

DH and SF wrote the manuscript. All authors contributed to the article and approved the submitted version.

## Conflict of Interest

The authors declare that the research was conducted in the absence of any commercial or financial relationships that could be construed as a potential conflict of interest.

## Publisher’s Note

All claims expressed in this article are solely those of the authors and do not necessarily represent those of their affiliated organizations, or those of the publisher, the editors and the reviewers. Any product that may be evaluated in this article, or claim that may be made by its manufacturer, is not guaranteed or endorsed by the publisher.

